# Feature extraction method for proteins based on Markov tripeptide by compressive sensing

**DOI:** 10.1186/s12859-018-2235-x

**Published:** 2018-06-18

**Authors:** C. F. Gao, X. Y. Wu

**Affiliations:** 1 School of Science, Jiangnan University, Wuxi, 214122 China; 2 Wuxi Engineering Research Center for Biocomputing, Wuxi, 214122 China

**Keywords:** Amino acid sequence, Proteins, Feature extraction, Compressive sensing, Markov transfer matrix

## Abstract

**Background:**

In order to capture the vital structural information of the original protein, the symbol sequence was transformed into the Markov frequency matrix according to the consecutive three residues throughout the chain. A three-dimensional sparse matrix sized 20 × 20 × 20 was obtained and expanded to one-dimensional vector. Then, an appropriate measurement matrix was selected for the vector to obtain a compressed feature set by random projection. Consequently, the new compressive sensing feature extraction technology was proposed.

**Results:**

Several indexes were analyzed on the cell membrane, cytoplasm, and nucleus dataset to detect the discrimination of the features. In comparison with the traditional methods of scale wavelet energy and amino acid components, the experimental results suggested the advantage and accuracy of the features by this new method.

**Conclusions:**

The new features extracted from this model could preserve the maximum information contained in the sequence and reflect the essential properties of the protein. Thus, it is an adequate and potential method in collecting and processing the protein sequence from a large sample size and high dimension.

## Background

Protein feature extraction is a key step to construct a predictor based on machine learning technique. Theoretically, the critical attributes within the protein can be obtained by extracting its features from amino acid sequences, then, by comparing the different features of proteins to predict the homologous biological function or identifying proteins for the localization of subcellular sites. Some software tools have been established to generate various protein features, such as Pse-in-One [1], BioSeq-Analysis [2], Pse-Analysis [3], etc. Pse-in-One is a powerful web server which covers 8 different modes to obtain protein feature vectors based on pseudo components. BioSeq-Analysis is a useful tool for biological sequence analysis which can automatically complete three steps: feature extraction, predictor construction and performance evaluation. Pse-Analysis a python package which can automatically complete five procedures: feature extraction, optimize parameters, model training, cross validation, and evaluation. These tools have been widely and increasingly used in many areas of computational biology. Since feature extraction is a necessary precondition for almost all existing prediction algorithms, the subsequent studies is based on the maximum retention of the protein attribute as assessed from the amino acid sequence.

The extraction of features for pattern recognition is challenging as a majority of the discriminant features are often difficult to find or cannot be measured due to some conditions that might complicate the feature extraction task. The initial sequences may be very large or complex that cannot be used directly without transformation in the process of identification, and therefore, we can use the projection method such that the sample data can be reduced to low-dimensional space. Thus, obtaining the maximum representative features of the nature of the characteristics is known as feature extraction [4].

Compressive Sensing (CS) established a new theory for signal processing based on sparse representation and optimization issue [5–7]. The CS theory transforms the sampling of a large number of sparse signals into that of a small amount of useful information while ensuring that crucial details are not destroyed. Previous studies found that when a signal is compressible or can be sparsely represented on a transform base, the high dimensional signal can be projected to a low-dimensional space through a measurement matrix (not related to the transform base). If the signal is sufficiently sparse, then it can be discriminative. Due to the excellent performance of the CS theory in collecting high-density information, it has been applied in other fields, and some new methods of feature extraction and recognition have been developed, including the classification algorithm based on sparse representation and its application in medical image [8], digital signal feature extraction [4], and video watermarking [9].

In order to acquire the effective and discriminative features of the protein, we used the sparse vector for feature representation of the protein sequence. The key idea is that the amino acid sequence is transformed into a sparse vector representation, followed by the extraction of the discriminating feature by the compression perception technique from the sparse vector.

## Methods

### Compressive sensing theory

**Fig. 1:**
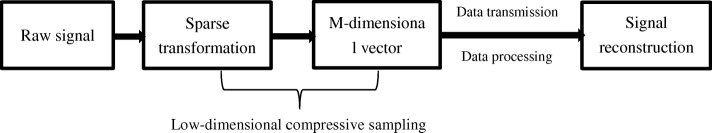
Block diagram of CS theory

Compressive Sensing (CS) theory is a new method of data acquisition by achieving the sparse signal. The CS theory discovered that when a signal is compressible or sparse in a transform domain, then a higher dimension sparse signal can be projected onto a lower dimension space with an appropriate measurement matrix, and the initial signal can be reconstructed by an optimized algorithm with a relatively high probability (Fig. 1).

Supposingx ∈ R^N^ is a one-dimensional signal of length *N*, which can be expanded by a set of orthogonal bases (sparse base) ψ, that is

1 $$ \mathrm{x}=\sum \limits_{i=1}^N{\psi}_i{\theta}_i=\psi \theta $$

Where ψ = [ψ_1_, ψ_2_, …ψ_N_] is a Ν × Ν matrix and ψ_i_ is a Ν × 1 vector. θ = {θ_1_,…,θ_N_} is a *N*-dimensional vector composed of N sparse coefficients θ_i_ = ψ_i_^T^x. If the signal *x* only contains *K* (*K* < <*N*) non-zero coefficients on the orthogonal basis *ψ*, then signal *x* is generally considered as sparse or compressible.

Consequently, signal *x* can be projected onto the measurement matrix Φ = {*ϕ*_1_, ⋯, *ϕ*_*m*_} to obtain the *M*-dimensional compressive vector of the signal *x*, which can be expressed as:

2 $$ s=\Phi \mathrm{x} $$

Where, Φ represents the Μ × Ν measurement matrix, and *s* represents the measurement vector of length *M*. The eq. (1) is substituted into eq. (2) to obtain.

3 $$ \mathrm{s}=\Phi \psi \theta =\Theta \theta $$

Herein, the original *N*-dimensional signal is reduced to the *M*-dimensional observation signal *s* (measured value) by projection. The Eq. (3) indicated that the measured value is the combined function of the original signal, which contains a small amount of high-density information from the entire original signal; thus, it is the optimal combination value of the original signal.

Notably, the measurement matrix Φ is required to meet the following conditions: the rows of the measurement matrix Φ, and the rows of the sparse matrix ψ cannot be represented by each other. In the current study, we selected a random matrix that follows the Gaussian distribution as a measurement matrix and can fulfill the requirements with high probability [10, 11].

### Feature extraction for proteins by CS

Since every protein is composed of a linear sequence of amino acids that are presented as symbolic sequences, it cannot be used as data for computerized analysis. Therefore, these symbol sequences are required to be translated into data sequence to obtain a digital feature vector. The purpose of feature extraction is to derive a valid mathematical expression of the sequence that can truly reflect the inherent properties of the protein. The projection process of CS can preserve the vital information and the structure of the signal; and therefore, CS theory is a promising and potential extraction method, which distinctly satisfies our requirements.

The Markov model is widely used in the analysis of biological data for finding new genes from open reading frames and predicting protein structures [12, 13]. Therefore, the processed amino acid sequence can be transformed into the sparse matrix by Markov chain model, and then, the sparse data can be projected by the CS theory, followed by extraction of accurate features.

#### Preparation of the data set

**Fig. 2:**
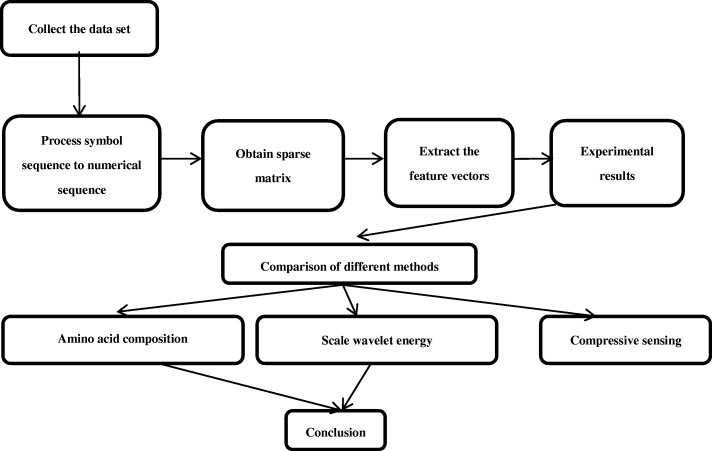
Block diagram of the experimental procedure

In the most abundant and most widely used protein database UniProt, we obtained a significant number of amino acid sequences according to the protein subcellular location, while constructing the experimental data set. The feature vectors were extracted by different methods: compressive sensing, amino acid composition, and scale wavelet energy. Finally, the different feature vectors extracted by each method are verified by Fuzzy C-means algorithm (FCM) for the corresponding classification accuracy (Fig. 2).

The standard data set used in the experiment is from the platform, http://www.uniprot.org, which is composed of three large databases of TrEMBL, Swiss-Prot, and PIR-PSD, wherein the data are characterized by high quality, no redundancy, and manual annotation for the protein sequence with high credibility and operational value.

The protein chain is commonly described as an amino acid sequence, and the element on the chain is the name of the amino acid. Suppose Ф is denoted as the basic character set of the 20 amino acids in alphabetical order, wherein each character represents a specific amino acid.

$$ \Phi =\left\{\mathrm{A},\mathrm{C},\mathrm{D},\mathrm{E},\mathrm{F},\mathrm{G},\mathrm{H},\mathrm{I},\mathrm{K},\mathrm{L},\mathrm{M},\mathrm{N},\mathrm{P},\mathrm{Q},\mathrm{R},\mathrm{S},\mathrm{T},\mathrm{V},\mathrm{W},\mathrm{Y}\right\} $$

Occasionally, in the current collected protein sequence, an unidentifiable amino acid (represented as the letter ‘X’) is present. The unknown specific amino acids will directly affect the subsequent sequence feature extraction. Thus, such sequence of the samples is removed automatically by the program, i.e., the sequences with letters not belonging to the set Ф are abandoned, in order to ensure the operational value and reliability of the sample and avoid cumbersome process of manual elimination in a large dataset.

Five hundred sequences of datasets of the nucleus and cell membrane were collected from the website based on the subcellular localization. Henceforth, this dataset is termed as A (Table 1) for convenience.

**Table 1:** Dataset A of 1000 Samples

Subcellular localization category	Number of samples
Nucleus	500
Cell membrane	500

The scale of datasets is further expanded, and nucleus (cell nucleus), cell membrane, and cytoplasm data are collected and labeled as dataset B (Table 2).

**Table 2:** Dataset B of 2400 Samples

Subcellular localization category	Number of samples
Nucleus	800
Cell membrane	800
Cytoplasm	800

#### Construction of Markov transfer matrices of protein sequences

The Markov model has a solid mathematical basis. The system transfer from one state to another is known as the Markov process. Essentially, it is a critical stochastic process and a mathematical model for the complex state transition. Markov chain is a collection of the state distributions. The amino acid sequences are commonly represented by a sequence of symbols that can be regarded as the Markov transition state, and the order between the symbols reflect the intrinsic relationship between the states. Thus, the amino acid sequence can be ascribed as a Markov process.

To ensure the sparseness of the Markov transfer matrix obtained from the protein, the state distribution of the transfer behavior of amino acids is described by a 20 × 20 × 20 frequency matrix M, where 20 types of amino acids are arranged in rows, columns, and longitudinally, respectively, followed by the construction of an adjacent matrix that reflects the composition of the tripeptide of the sequence.

Supposing L_i, j, k_ = {(*X*, *Y*, *Z*, p)} denotes the adjacent relationship of the tripeptide ‘*XYZ’,* wherein p is the occurring frequency of segments ‘*XYZ*’ throughout the sequence. We assigned

4 $$ \mathrm{M}\left(\mathrm{i},\mathrm{j},\mathrm{k}\right)=\mathrm{p} $$

Wherein the i^th^ row corresponds to amino acid X and the j^th^ column corresponds to Y, while the k^th^ longitudinal corresponds to Z. All the existing tripeptides were searched in the protein sequence and the corresponding values assigned in matrix M. Consequently, the information about the intrinsic relation of the protein is shown to satisfy the sparse conditions of the CS theory, i.e., the Markov’s transfer frequency matrix.

**Fig. 3:**
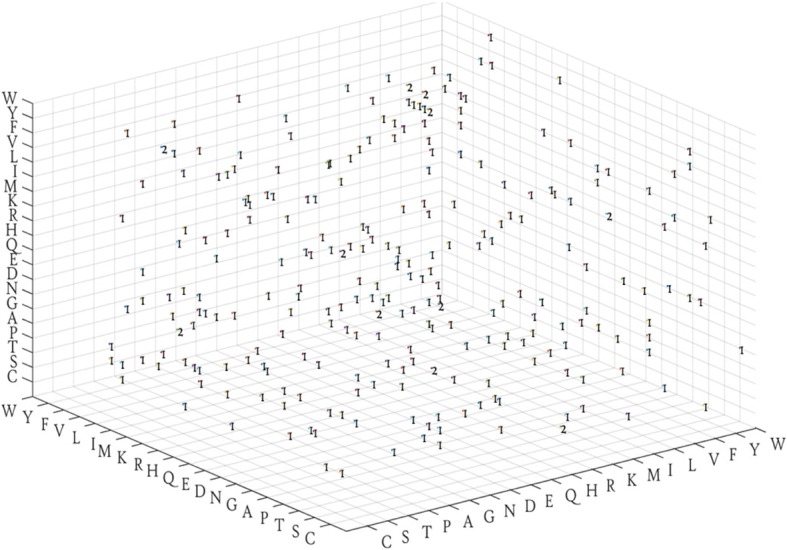
Three-dimensional Markov frequency matrix of ZIG1_CAEEL

The following is a protein sequence, whose subcellular localization is cell membrane and Swiss-Prot ID is ZIG1_CAEEL (Table 3). The sequence is converted into Markov frequency matrix (Fig. 3).

**Table 3:** Sample information of ZIG1_CAEEL

Entry	Length	Sequence	Subcellular location
G5EGI7	265	MKNLLLITFFVVSTVTALGGRGSKSALVLVAARSSENHPLHATDPITIWCAPDNPQVVIKTAHFIRSSDNEKLEAALNPTKKNATYTFGSPSVKDAGEYKCELDTPHGKISHKVFIYSRPVVHSHEHFTEHEGHEFHLESTGTTVEKGESVTLTCPVTGYPKPVVKWTKDSAPLALSQSVSMEGSTVIVTNANYTDAGTYSCEAVNEYTVNGKTSKMLLVVDKMVDVRSEFQWVYPLAVILITIFLLVVIIVFCEWRNKKSTSKA	SUBCELLULAR LOCATION: Cell membrane {ECO:0000305}; Single-pass type I membrane protein {ECO:0000305}.

The Markov frequency matrix is a three-dimensional square matrix. The elements in the matrix are integers (representing the frequency that the state transition actually occurs), different from the probability matrix (the elements are the decimal numbers within [0,1]). If the elements in the Markov frequency matrix are divided by the sum of the elements of the matrix, then they could be transformed into a Markov probability matrix and would possess all the properties of the Markov probability matrix. For convenient description, we used the shortened form of the Markov matrix for Markov transition frequency matrix in the following evaluations.

#### Extraction features from proteins by CS

Since the integers in the matrix represent the frequency of the three adjacent amino acids, the non-zero value would not exceed L-2, where L is the length of the protein. Thus, the Markov matrix harbors a crucial characteristic of sparseness, which is consistent with the property of sparse signal (relative to the signal length, only a few coefficients are non-zero, and the remaining is primarily zero).

The Markov matrix is expanded to obtain a one-dimensional vector x with length 8000 (L < < 8000) and the signal x is sufficiently sparse, such that the unit orthogonal matrix can be sued directly as the sparse base. As mentioned in section “Methods”, we selected independent and identically distributed Gaussian Random matrix (denoted byΦ) as the measurement matrix for the compressive projection. The inner product obtained by Eq. (3) was the low-dimensional observation signal s, which was the extracted feature set of the protein.

In Fig. 4:

**Fig. 4:**
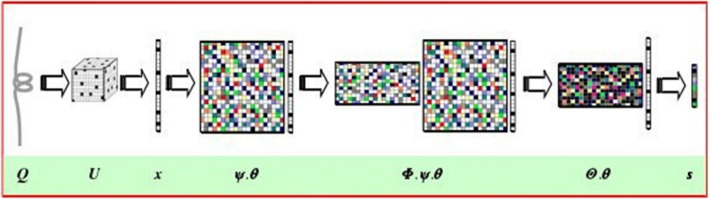
Schematic diagram of the feature extraction method by CS

**Table 4:** Indicators of three features on dataset A by different methods

Feature extraction method	Identification indicator		
	Accuracy	*Etp*	*tr*(*S*_*w*_)/*tr*(*S*_*b*_)
Compressive Sensing	0.8460	0.225	7.83
Amino acid composition	0.7130	0.999	13.11
Scale wavelet energy	0.8400	0.252	8.71

Q is the initial amino acid sequence;

U is a 20 × 20 × 20 three-dimensional Markov transfer frequency matrix;

x is an expanded one-dimensional sparse signal with length 8000;

ψ: is an 8000 × 8000 sparse base;

θ: is the conversion of the signal x under sparse base *ψ*;

Φ is a m × 8000 measurement matrix;

s is the compressed measurement signal with the length of m, and s indicates the extracted protein features.

The advantage of the CS method is that the sparse signal can be compressed while reflecting the transfer behavior in the Markov matrix. Thus, the low-dimensional measurement signal s indicates the high-density features and maintains the structure information adequately; this is precisely as expected of the intrinsic properties of the protein.

## Results and discussion

**Fig. 5:**
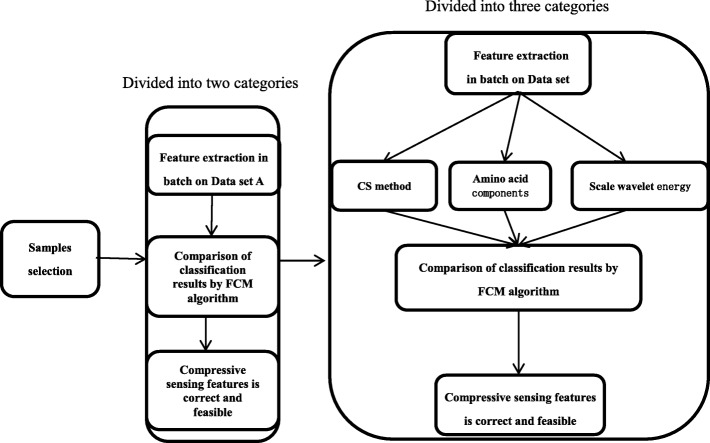
Schematic of the verification with different features

Dataset A is divided into two types according to the subcellular location, and the feature vectors are extracted in batches. Subsequently, the classification accuracy by FCM algorithm is calculated in order to examine whether the CS method is correct and feasible. Furthermore, we extracted the feature vectors from the amplified dataset B by CS method, amino acid composition, and scale wavelet energy, and these features were verified by FCM algorithm. The comparison results suggested that the feature extracted by CS was superior to the other methods (Fig. 5).

### Evaluation indexes for the feature set

#### Effectiveness

The validity of the features needs to be tested by specific indexes, especially comparison of the features of scale wavelet energy and amino acid composition. In this case, the following indicators were used in the experiments. The criteria were as follows: the intraclass distance as small as possible and the interclass distance as large as possible.

5 $$ Sw=\sum \limits_{k=1}^C\sum \limits_{i=1}^{Nk}\left({\boldsymbol{x}}_i^{(k)}-{\boldsymbol{m}}_k\right){\left({\boldsymbol{x}}_i^{(k)}-{\boldsymbol{m}}_k\right)}^T $$

6 $$ {S}_b=\sum \limits_{k=1}^C{N}_k\left({\boldsymbol{m}}_k-\boldsymbol{m}\right){\left({\boldsymbol{m}}_k-\boldsymbol{m}\right)}^T $$

Where *C* is the class number, *N*_*k*_ is the number of samples in the k^th^ class, *m*_*k*_ is the mean vector of the k^th^ class, *m* is the mean vector of all samples, *tr*(*S*_*w*_) is the intra-class distance, *tr*(*S*_*b*_) is the interclass distance, and the smaller the ratio *tr*(*S*_*w*_)/*tr*(*S*_*b*_), the better the recognition effect.

#### Entropy function

Entropy can be used to evaluate the performance of the features of different species and present the percentage of all those identified accurately. The entropy function is defined as:

7 $$ Etp=-\frac{1}{\mathrm{n}}\sum \limits_{k=1}^C\sum \limits_{i=1}^n{u}_{ik}{\log}_2\left({u}_{ik}\right) $$

Where n is the number of samples in a given dataset, C is the number of clusters and u_ik_ represents the membership of the i^th^ sample belonging to k^th^ class, and accordingly, the smaller the Etp value, the better the clustering effect.

#### Clustering accuracy

FCM algorithm is widely used in pattern recognition, whereby the clustering performance is adequate. Compared to the other common recognition algorithm, FCM is a more efficient and rapid data analysis method, such that it can be selected objectively for the clustering accuracy test.

Since datasets A and B are collected from UniProt database according to the subcellular localization, the actual categories of the dataset have been determined in advance. Then, the accuracy of the clustering results is calculated, i.e., the ratio between the correctly recognized sample size and the total sample size to assess the effect of classification and compare the discriminative effect of different methods of feature extraction. We defined the clustering accuracy as:

8 $$ A\mathrm{ccuracy}=\frac{1}{N}\sum \limits_{\mathrm{k}=1}^C\sum \limits_{\mathrm{i}=1}^{n_k}{x}_{ik} $$

Where, N is the total number of samples in the dataset, C is the number of clusters, *n*_*k*_is the actual sample size in the k^th^ class, and *x*_*ik*_ represents the two-value clustering result of the i^th^ sample in the k^th^ class (if the classification is correct, then the value is 1, or else 0).

### Recognition results and analysis of features

#### Recognition results of dataset A

Dataset A was collected according to the subcellular localization (nucleus and cell membrane, Table 1) that can be categorized into two classes by FCM algorithm. The features of dataset A are extracted by three methods and the corresponding indicators as shown in Table 4.

**Fig. 6:**
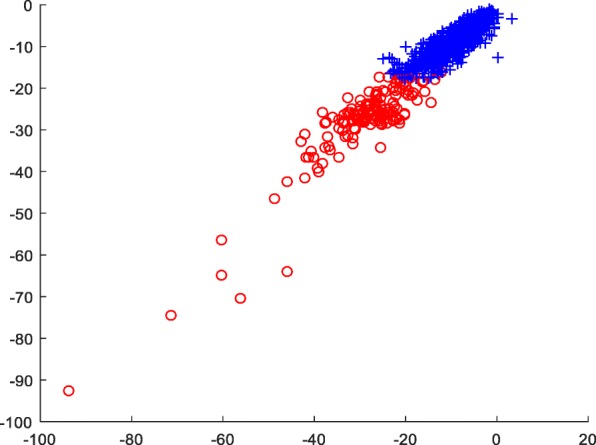
Two-dimensional distributions of CS features on dataset A

For the sample size of 1000 with two categories, the result of compression perception was optimal. In order to intuitively observe the distribution of the features extracted by the CS method and maintain the distance between the original samples considerably, we used linear mapping [14] to project the extracted CS feature vector into a two-dimensional plane. Thus, the distribution of two proteins was distinguishable (Fig. 6).

**Fig. 7:**
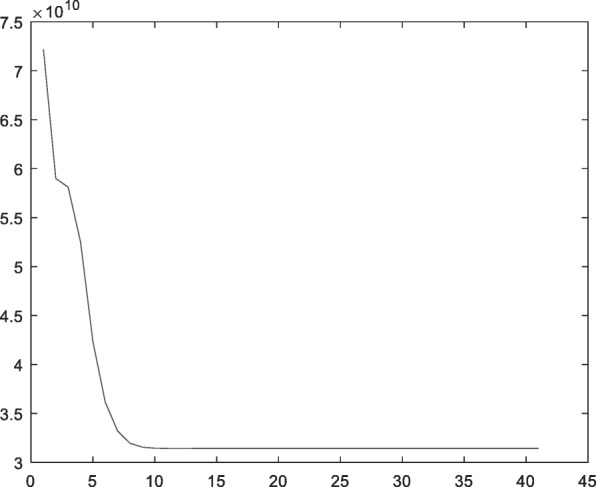
Variation of objective function of FCM algorithm on dataset A

Consecutively, the convergence of the objective function of FCM algorithm with the CS features was satisfactory (Fig. 7), and the results demonstrated the reasonability of FCM algorithm in the current experiment.

**Table 5:** Indicators of the three features on dataset B by different methods

Feature extraction method	Identification indicator		
	Accuracy	*Etp*	*tr*(*S*_*w*_)/*tr*(*S*_*b*_)
Compressive Sensing	0.7588	0.392	2.092
Amino acid composition	0.6946	1.585	1.306
Scale wavelet energy	0.7125	0.460	2.014

#### Recognition results of dataset B

Based on dataset A, the effect of the two methods of compression sensing and scale wavelet energy did not vary significantly (Table 4), which could be attributed to the small sample size. Furthermore, dataset B (subcellular localization for the nucleus, cell membrane, and cytoplasm) was collected by amplifying the capacity of the dataset and subcellular localization categories. The identification result of dataset B is shown in Table 5.

When the category and the sample size increases, the complexity of data analysis increases. Consequently, the effective indicators based on Eqs. (5, 6, 7 and 8) of the three methods have declined. However, Table 5 demonstrated that the clustering effect based on the CS features continued to be superior to the amino acid composition and the scale wavelet energy features. Thus, the feature extraction method by CS was optimal.

**Fig. 8:**
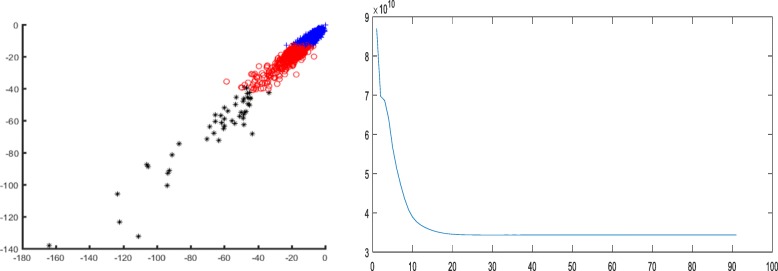
Classification result and objective function variation with CS features on dataset B

**Fig. 9:**
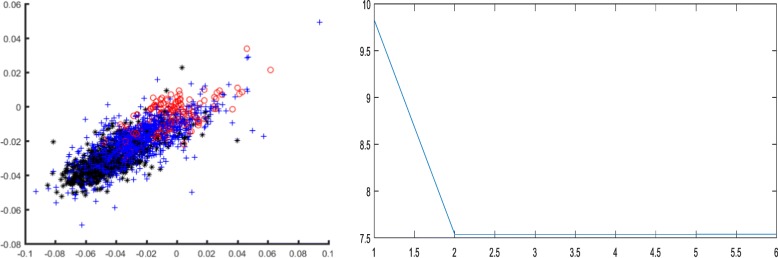
Classification result and objective function variation with amino acid composition features on dataset B

**Fig. 10:**
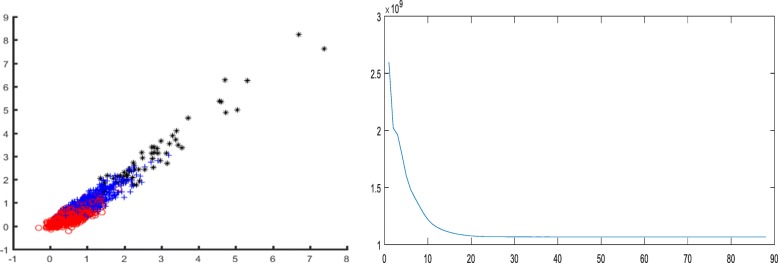
Classification result and objective function variation with scale wavelet energy features on dataset B

The executions of several previous identification algorithms required an additional prior knowledge of training samples. Nevertheless, the method in this study can achieve the relatively high recognition accuracy in the case of unsupervised clustering without any training samples, which reflects the advantages of CS theory in collecting vital information. In order to intuitively illustrate the effect of each feature extraction method, the clustering results are shown in Figs. 8, 9, and 10.

**Table 6:** Indicators of CS features with different dimensions on dataset A

Feature vector	*tr*(*S*_*w*_)*/ tr*(*S*_*b*_)	Clustering accuracy
5 - dimensional CS features	7.83197	0.8460
10 - dimensional CS features	7.82934	0.8460
15 - dimensional CS features	7.82953	0.8460
20 - dimensional CS features	7.83036	0.8460
30 - dimensional CS features	7.83051	0.8460
50 - dimensional CS features	7.83031	0.8460

Figures 8, 9 and 10 demonstrated that the recognition effect with CS features was better than the others. In accordance with the theoretical analysis in section “Methods”, the CS theory exhibited a great advantage in the collection of critical information to obtain the discriminative features. On the contrary, amino acid composition features showed excessive overlap resulting in unsatisfactory recognition with mispartition.

### Compression scale analysis

We used dataset A to investigate the relationship between the compression scale of the measurement matrix (i.e., the dimension of the feature vector after extraction) and the effect of feature expression.

Table 6 compared the features with different compressive dimensions, the distance between the class, and the distance within the class, and only slight differences were observed. The small difference in the clustering validity index arose from the randomness of the measurement matrix; however, it did not affect the clustering accuracy. The results in Table 6 suggested that the CS features were not sensitive to the dimension of the measurement matrix.

### Methodological discuss

The Markov transfer frequency matrix contains both the number of residues and the order of sequence and also reflects the intrinsic structural information. Altogether, it can be regarded as the synthesis method of the amino acid component [15], the sequence order method [16], and the wavelet decomposition method [17]. Therefore, the feature extracted by the CS method showed better robustness in the experiments as compared to the other two traditional methods based on the fact that feature is extracted from the same sample data set using the scale wavelet energy and the amino acid component, respectively.

Since our methods is focus on the expression formulate of sequence and feature extraction, consequently it is suitable for discriminative prediction models. Besides the recognition of proteins subcellular localization in current study, an important and suitable task in protein sequences analysis and/or performance evaluation is remote homology detection, e.g. protein features representation can be combined to improve the sensitivities of predictors [18], discriminative models and ranking approaches are complementary for the improvement of predictive performance [19]. These ideas in protein remote homology detection would provide a promising direct for future research.

## Conclusions

The CS theory can capture sufficient information while a sparse signal is compressed, and the projection vector is an excellent discriminant which is the combination function of the sparse signal. In the present study, this theory is introduced to develop a new feature extraction method of the protein sequence. Herein, the amino acid frequency, the order of the sequence, the structure, and other vital information of the protein is transferred into a sparse signal by the Markov transfer matrix, and then, the accurate feature expressions are extracted from the sparse vector by CS theory.

The new bioinformatics theoretical framework of protein is constructed based on the Markov model and the theory of compression sensing. It is an adequate feature extraction method in collecting and processing the protein sequence with large sample size and high dimension. Moreover, it is suitable for the development of biological information processing and has the potential of extension and application in several other fields [20, 21]. However, there is yet room for improvement in this method with respect to the following aspects:

1. The Markov transfer frequency matrix used in our method is excellent and feasible; however, it is not the sole method to quantify the amino acid sequence of the protein, and other methods can be attempted to quantify the symbol sequence in the future. In addition, if the measurement matrix can satisfy the adaptive requirements according to the observation data, the compressive performance of the CS technology would be improved further.
2. Several investigations to the CS theory are primarily focused on the fixed orthogonal space. Consecutively, finding the sparse domain of the signal is a critical prerequisite for the application of the CS theory. Several studies have shown that the sparse representation of the signal is effective under the super-complete redundancy dictionary. Interesting studies in this area have made some progress, which would provide a promising direction for future exploration in terms of improvement in the method.

## Abbreviations

CS: Compressive sensing
FCM: Fuzzy C-means algorithm
